# Hypoxia refines plasticity of mitochondrial respiration to repeated muscle work

**DOI:** 10.1007/s00421-013-2783-8

**Published:** 2013-12-11

**Authors:** Dominique Desplanches, Myriam Amami, Sylvie Dupré-Aucouturier, Paola Valdivieso, Silvia Schmutz, Matthias Mueller, Hans Hoppeler, Roland Kreis, Martin Flück

**Affiliations:** 1 Institute for Biomedical Research into Human Movement and Health, Manchester Metropolitan University, Manchester, UK; 2 Centre de Génétique et de Physiologie Moléculaire et Cellulaire, CNRS UMR 5534, Université Lyon 1, Villeurbanne, France; 3 Institute of Anatomy, University of Bern, Bern, Switzerland; 4 Department of Clinical Research, University of Bern, Bern, Switzerland; 5 Laboratory for Muscle Plasticity, Department of Orthopaedics, Balgrist University Hospital, University of Zurich, Forchstrasse 340, 8008 Zurich, Switzerland

**Keywords:** Exercise, Oxygen, Metabolism, Plasticity, Cytochrome *c*, Gene

## Abstract

**Purpose:**

We explored whether altered expression of factors tuning mitochondrial metabolism contributes to muscular adaptations with endurance training in the condition of lowered ambient oxygen concentration (hypoxia) and whether these adaptations relate to oxygen transfer as reflected by subsarcolemmal mitochondria and oxygen metabolism in muscle.

**Methods:**

Male volunteers completed 30 bicycle exercise sessions in normoxia or normobaric hypoxia (4,000 m above sea level) at 65 % of the respective peak aerobic power output. Myoglobin content, basal oxygen consumption, and re-oxygenation rates upon reperfusion after 8 min of arterial occlusion were measured in *vastus* muscles by magnetic resonance spectroscopy. Biopsies from *vastus lateralis* muscle, collected pre and post a single exercise bout, and training, were assessed for levels of transcripts and proteins being associated with mitochondrial metabolism.

**Results:**

Hypoxia specifically lowered the training-induced expression of markers of respiratory complex II and IV (i.e. SDHA and isoform 1 of COX-4; COX4I1) and preserved fibre cross-sectional area. Concomitantly, trends (*p* < 0.10) were found for a hypoxia-specific reduction in the basal oxygen consumption rate, and improvements in oxygen repletion, and aerobic performance in hypoxia. Repeated exercise in hypoxia promoted the biogenesis of subsarcolemmal mitochondria and this was co-related to expression of isoform 2 of COX-4 with higher oxygen affinity after single exercise, de-oxygenation time and myoglobin content (*r* ≥ 0.75). Conversely, expression in COX4I1 with training correlated negatively with changes of subsarcolemmal mitochondria (*r* < −0.82).

**Conclusion:**

Hypoxia-modulated adjustments of aerobic performance with repeated muscle work are reflected by expressional adaptations within the respiratory chain and modified muscle oxygen metabolism.

## Introduction

Repeated endurance work (i.e. training) increases the capacity for aerobic ATP production in skeletal muscle through an elevation in the volume density of mitochondria in untrained subjects (Hoppeler et al. 1985). This local adaptation is specifically modified by the ambient concentration of oxygen during the workout. With bicycle-type endurance training under lowered oxygenation (hypoxia; Desplanches et al. 1993; Schmutz et al. 2010; Vogt et al. 2001), this is reflected by larger increases in the volume density of subsarcolemmal mitochondria compared to those residing between myofibrils. Concomitantly, we identify specific improvements in maximal oxygen uptake (*V*O_2max_), aerobic power, and fatigue resistance with a single bout of exercise in hypoxia after endurance training in hypoxia compared to normoxia (Hoppeler and Desplanches 1992; Ponsot et al. 2006; Zoll et al. 2006).

Subsarcolemmal mitochondria demonstrate shorter diffusion distances to capillaries than intermyofibrillar mitochondria (Kayar et al. 1988) and are thought to enhance the availability of oxygen for mitochondrial respiration. Accordingly, an increased volume density of the subsarcolemmal subpopulation of mitochondria, or an increased capillary-to-fibre ratio (Desplanches et al. 1993), would offer a distinct metabolic advantage for muscle working aerobically when the oxygen supply to the muscle is diminished, as occurs during hypoxaemia. The benefit of this adaptation is illustrated for the flight muscle of bareheaded geese, which migrate over the Himalayas (Scott et al. 2009).

**Fig. 1:**
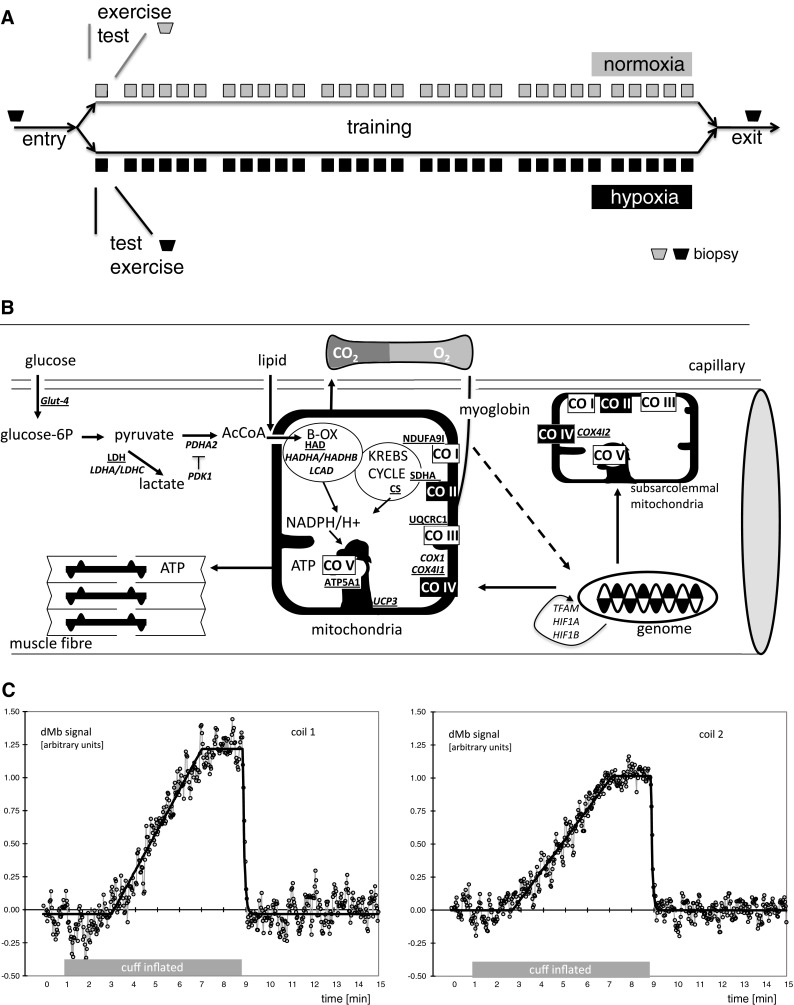
Experimental design and assessed parameters. **a** Scheme depicting the outline of the experiments. For details see the “Methods” section. Subjects experienced an entry test during which anthropometry and oxygen metabolism in *m. vastus lateralis* at rest, and aerobic performance in hypoxia or normoxia were assessed, and a resting muscle biopsy was collected from *vastus lateralis*. A week later subjects carried out an endurance exercise test at the respective training condition (i.e. warm-up followed by 30-min bicycle exercise at 65 % of the respective *P*_max_) during which biopsies were collected 24 h after the exercise bout. Subjects then entered an endurance-training protocol with 30 exercise sessions of 30-min on a stationary bicycle at 65 % *P*_max_ in normoxia or normobaric hypoxia equivalent to 4,000 m above sea level. This was followed by a post-training biopsy and an exit test essentially repeating the entry test. **b** Scheme depicting the localization of assessed factors of mitochondrial metabolism in muscle fibres. *Continuous arrows* indicate the flow of metabolic processes. *Boxes highlighted by black filling with text in white font* reflect the two respiratory complexes where hypoxia-dependent regulation is identified. Protein and transcript species being assessed are indicated with *underlined font* and in *italics*, respectively. *AcCoA* acetyl coenzyme A, *B-OX* beta oxidation, *CO I*–*CO V* complex I to complex V of the mitochondrial respiration chain. Further names are defined in the list of abbreviations. **c** dMb signals from 2 independent receive coils as determined by 1H MRS in function of time and as consequence of applying a blood pressure cuff placed on the thigh in a one subject before the start of exercise. *Circles and the connecting grey line* represent data points filtered as a moving average of 3 original fitted points. The *black line* is the result of fitting this signal with a model for a delayed linear increasing signal, followed by a plateau and an exponential recovery upon release of the air cuff

Today, the relevance of an increased volume density in subsarcolemmal mitochondria for respiration in skeletal muscle and the molecular factors underpinning the hypoxia-specific biogenesis of subsarcolemmal mitochondria is not well understood (Elustondo et al. 2013). For instance, subsarcolemmal mitochondria and their distribution respective to capillaries are largely independent of the level of coarse factors associated with aerobic metabolism, such as citrate synthase, 3-hydroxyacyl-CoA dehydrogenase, and the myocellular oxygen carrier myoglobin (Kayar et al. 1988). As well, the general increase of mitochondrial gene transcripts in recruited muscle during recovery from exercise cannot explain the specific increase in subsarcolemmal mitochondria with endurance training in hypoxia (Fluck 2006; Perry et al. 2010; Pilegaard et al. 2000; Schmutz et al. 2010; Wagner 2012). Candidate factors involved in the hypoxia-specific improvement of local aerobic capacity with endurance training include those that operate at critical biochemical steps of mitochondrial respiration under lowered muscle oxygenation with intense exercise in hypoxia (Fig. 1b; Flueck 2009; Richardson et al. 1995). This situation is indicated for the oxygen-sensitive cytochrome *c* oxidase. For this respiratory chain component, a switch in expression from isoform one of subunit 4 (COX4I1) towards isoform two (COX4I2) with higher affinity for oxygen is observed in culture upon exposure to ambient hypoxia (Fukuda et al. 2007). Possibly, the adjustments comprise altered coupling of oxidative phosphorylation and ATP synthesis in mitochondria through modified expression of UCP3, as this is shown to affect the efficiency of muscle work (Mogensen et al. 2006). Mitochondrial adjustments to repeated exercise in hypoxia may also connect to metabolic reactions, which improve the generation of high-energy phosphates at a reduced reliance on oxygen (reviewed in Green et al. 2009). This may comprise an increased capacity of the citrate cycle as indicated by elevated activity of citrate synthase after endurance training in hypoxia (Desplanches et al. 1993). As well, mitochondrial metabolism may be increasingly fuelled by pyruvate arising from the conversion of glucose after its contraction-induced import in muscle fibres (Fluckey et al. 1999), and through the conversion of lactate by the mitochondria-associated isoform C of lactate dehydrogenase (Andrade and McMullen 2006; Elustondo et al. 2013; Horowitz et al. 2005; Zoll et al. 2006).

To further the understanding of hypoxia-modulated muscle adaptation, we assessed whether adjustments in subsarcolemmal mitochondria and muscle oxygenation in the knee extensor *m. vastus lateralis* with bicycle-type endurance training would be associated. In vivo information about muscle oxygenation, including basal oxygen consumption rate, perfusion and myoglobin content, was obtained by ^1^H MR spectroscopy via the quantification of the deoxymyoglobin (dMb) signal during and following arterial occlusion (reviewed in Carlier et al. 2006; Baumgartner et al. 2005; Richardson et al. 2001; Tevald et al. 2009). Furthermore, we hypothesized that hypoxia-specific level adjustments of selected gene transcripts after a single bout of bicycle-type endurance exercise and their respective proteins after training would manifest in the recruited muscle, *m. vastus lateralis*, and demonstrate correlative relationships to changes in cellular and functional variables of mitochondrial respiration. A time point 24 h after one bout of endurance exercise was selected to monitor hypoxia-specific adaptations of transcript expression because the switch in COX4 isoform expression occurs 24 h after a hypoxic stimulus in culture (Fukuda et al. 2007) and because this reflected the situation when subjects would carry out a next bout of exercise during their daily training.

## Methods

### Experimental design

Twelve young, healthy male volunteers consented to participate in this study after being fully informed about the possible risks. Following an entry test during which anthropometry and oxygen metabolism at rest in *vastus lateralis* were assessed, a muscle biopsy was collected from *vastus lateralis* muscle after 3 days without vigorous physical activity (Fig. 1a). Subsequently subjects were assigned to either of two groups, training in normoxia or hypoxia (*n* = 6 each). They performed *V*O_2max_ tests to assess aerobic performance in hypoxia or normoxia. After 7 days of rest they carried out an endurance exercise test composed of a single bout of endurance exercise at the respective training condition, i.e. normoxia or normobaric hypoxia equivalent to 4,000 m above sea level. Further biopsies were collected 24 h after the exercise bout and subjects then began within the next 7 days a training protocol in hypoxia or normoxia at 65 % of *P*_max_ in hypoxia or normoxia, respectively. This was followed by a post-training biopsy and the repetition of the *V*O_2max_ tests in normoxia or hypoxia. The study protocol has been approved by the ethics committee of the Canton of Bern (Switzerland) and has been performed in accordance with the ethical standards laid down in the 1964 Declaration of Helsinki. Extracts of the data (anthropometry, exercise performance and the consequences of single exercise and training on selected muscle morphometric parameters (volume density of mitochondria and capillarity) have been previously reported (Schmutz et al. 2006, 2010). In this study, an additional piece of biopsy material was processed for further molecular characterization. This comprised transcript profiling of an array of gene transcripts involved in regulation of mitochondrial metabolism prior and post the single bout of exercise and the measure of changes in protein levels in immunoblots or activity in enzymatic assays along with morphometric estimation of cellular variables pre- and post-endurance training (Fig. 1b).

### Anthropometry

Anthropometric parameters (age, height, body mass and body fat percentage) were determined at the beginning of the study and after 6 weeks of training in hypoxia. Body fat percentage was determined by a seven-point skin-fold measurement using a calibrated calliper (GPM, Zurich, Switzerland).

### ^*1*^*H*-*MRS based measures of muscle oxygen metabolism*

The features of the ^1^H-MRS methodology and its evaluation were described in detail in (Kreis et al. 2001) and (Baumgartner et al. 2005). In the present work, the dMb signal was acquired from the thigh, rather than the calf. In short, pulse and acquire spectra (110 ms repetition time, semi-selective excitation pulse) were acquired on a 1.5 T MR scanner (Signa, General Electric, Milwaukee, USA) using two small independent surface receive coils (7.5 cm diameter) reproducibly placed in a semi-rigid coil holder such as to be most sensitive to vastus medialis muscle in mid-thigh and 3 cm more distally to vastus lateralis muscle. The exact position of the coil relative to the muscle was dependent on the size of the volunteer’s thigh. The coil was placed on the same location in the pre- and post-training acquisition using external anatomical markers and controlled with internal anatomical markers. The dMb signal was induced by inflating a standard blood pressure cuff placed on the thigh as far proximal as possible to 220 mmHg to ensure complete arterial occlusion (Fig. 1c). Inflation of the cuff and its release were synchronized to the MR acquisition using an automatically paced air pressure and release system. Total acquisition time was 15 min 18 s. First, a baseline signal of dMb (56 s) and water (1 shot with pre-delay for full signal of 5 s) were recorded before the cuff was automatically inflated and kept inflated for 8 min. After deflation of the cuff, signal acquisition continued to monitor signal disappearance for 6 min 13 s. Further fully recovered reference signals from water were intermittently recorded just before pressure release and at the end of the recovery. Automatic data processing was done with the manufacturer’s spectroscopy package (SAGE) and using the fitting program TDFDfit (Slotboom et al. 1998). Quantitation of the dMb signal was based on the water signal recorded from the same volume as the dMb signal and an assumed water content. The following parameters were extracted: [Mb], myoglobin content in mmol/kg; BOCR, basal oxygen consumption rate (ml O_2_/kg); DT, de-oxygenation time − the time in minutes to reach complete de-oxygenation of Mb; ORT, oxygen repletion time − a characteristic time in seconds for oxygen repletion upon release of arterial occlusion (inverse of exponential rate constant).

### *V*O_2max_ tests

Maximal oxygen uptake and peak aerobic power output in normoxia or hypoxia was assessed with ergospirometry on a stationary bike (Ergoline 800S, Ergoline GmbH, Bitz, Germany) as described (Schmutz et al. 2010).

### Endurance exercise test

A single bout of endurance exercise consisting of a two-step protocol on the Ergoline 800S ergometer in the respective oxygen condition was carried out as follows. After a warm-up period of 10 min at a low intensity (40 % *P*_max_), the subjects cycled for 30 min at 65 % *P*_max_ in the respective oxygenation condition during training. Arterial oxygen saturation and lactate concentration at the fingertip were assessed as described previously (Schmutz et al. 2010).

### Endurance training

Subjects completed a protocol of 30 endurance exercise sessions of 30 min on a stationary bicycle (Kettler, Ense-Parsit, Germany) in the assigned condition, i.e. normoxia at 560 m above sea level or normobaric hypoxia at a simulated altitude of 4,000 m above sea level, at 65 % of corresponding *P*_max_ in normoxia and hypoxia, respectively. Intensity of the individual exercise bouts was controlled and adjusted based on daily heart rate and weekly lactate measures. For the hypoxia and normoxia group the subjects completed 89 and 92 % of the 30 possible training sessions, respectively.

### Tissue handling

Biopsies were collected and stored in liquid nitrogen as described previously (Schmutz et al. 2010). Frozen specimens were transported, if necessary, in dry ice in insulated boxes (Carbagas, Liebefeld, Switzerland).

### Transcript profiling

Total RNA was isolated, reverse transcribed and subjected to polymerase chain reaction using SYBR Green-based chemistry or custom microarray analysis as reported and deposited under accession codes GSE 13623 and GSE 2479, respectively, at GEO (Schmutz et al. 2010). Raw values were normalized to 28S RNA. The expression level of 15 gene transcripts associated with mitochondrial metabolism (COX1, COX4I1, COX4I2; HADHA, HADHB, LCAD, UCP3), lactate and pyruvate metabolism (LDHA, LDHC, PDHA2, PDK1) regulation of mitochondrial transcript expression (TFAM, HIF1A, HIF1B) and glucose uptake (GLUT4) were measured. The COX4I2 measures were carried out with QuantiTect primer assay Hs_COX4I2_1 SG (Qiagen).

### Enzymatic assays

Muscle homogenates were prepared in 0.3 M phosphate buffer containing 0.05 % bovine serum albumin (pH 7.7) and assayed spectrophotometrically for the activity of HADH (EC 1.1.1.35), LDH (EC 1.1.1.27) and citrate synthase (CS; EC 4.1.3.7) as described (Desplanches et al. 1987).

### Immunoblots

The protein expression levels of each of the five respiratory complexes in mitochondria, i.e. NDUFA9 (complex I), SDHA (complex II), UQCRC1 (complex III), COX4I1 and COX4I2 (complex IV), complex V (ATP5A1), and GLUT4 were assessed in immunoblots using a standard protocol with a paired design. In each lane 20 μg of protein was loaded, essentially as described before (Flueck et al. 2011; Giraud et al. 2005). In brief, muscle homogenates in phosphate buffer were diluted in SDS-loading buffer at 1 mg/ml, denatured for 5 min at 95 °C and proteins separated via 10 % SDS-PAGE and western blotted onto nitrocellulose membrane (Protean, Amersham). Nitrocellulose membranes were stained with Ponceau S to verify equal loading in all lanes and visualize the band corresponding to actin. Subsequently immunodetection was carried out for the six mitochondrial proteins simultaneously using a commercial mix of monoclonal antibodies (Anti-OxPhos Complex Kit, Molecular Probes/Invitrogen Ltd, Paisley, UK), COX4I2 (clone 1F2, purified immunoglobulin, SIGMA #WH0084701M1), UCP3 (Millipore, #AB3046) and GLUT4 (Millipore, #07-1404). Signal detection was carried out with enhanced chemoluminescence (Femto kit, Pierce) and quantified with a Chemidoc system running under Quantity One software (Bio-Rad, Life Science Research, Hercules, CA, USA). The signal intensity of the respective band was estimated with the ‘volume rectangular tool’ and corrected for background of a band of equal height and size (area) in an empty sample lane. Background-corrected data were normalized to actin and then normalized to the mean values of the samples before training for the respective gel. The values therefore reflect relative expression levels per total muscle protein.

### Morphometry

Variables of muscle composition such as fibre cross-sectional area (CSA) (in μm^2^), capillaries per fibre, capillaries per unit fibre area (mm^−2^), and volume densities of organelles in muscle fibres (%), i.e. densities of intermyofibrillar mitochondria, subsarcolemmal mitochondria, total mitochondria, intramuscular lipid, sarcoplasm) were assessed in muscle biopsies as described previously (Schmutz et al. 2010). The CSA of muscle fibres was assessed with modifications as described in Durieux et al. (2009). Twelve-micrometer-thick cryosections were prepared from muscle biopsies and subjected to fibre typing using mouse anti-type II MHC (Sigma Chemical, Buchs, Switzerland), and horseradish peroxidase-coupled secondary anti-mouse antibody (Cappel, ICN Biomedicals). The immunohistochemical signal was digitally recorded using an Olympus IX50 microscope with digital camera DP72 (Olympus Schweiz AG) being operated with the CellSens Dimension software. Three random fields were recorded at a 10× magnification for each section. Image processing of microscopic fields was performed using Adobe Photoshop CC (Adobe Systems Incorporated). Fibres were assigned to the respective fibre type based on the immunohistochemical staining (i.e. type I for non-stained fibres and type II for the stained fibres). Subsequently, the circumference of muscle fibres that met the criteria of being presented without significant signs of distortion or folding (e.g. circularity factor > 0.7) was assessed with the lasso tool and the CSA and distribution of type I and type II fibres was imputed. The scale of each image was calibrated by a horizontal bar on the image as inserted by the software and adjusting with pixel-length. On average, 56 type I and 60 type II muscle fibres were counted per muscle biopsy and time point of training. Image analyses were performed by two co-authors (PV and MF).

### Statistics

Interaction effects were assessed with a repeated-measures ANOVA for the repeated factors ‘exercise’ (i.e. pre/post) or training (i.e. pre/post) and the between-factor ‘co-stimulus’ (i.e. normoxia/hypoxia) (Statistica 10.0, Statsoft). Changes post vs. pre exercise and endurance training, respectively, were subsequently assessed with a post hoc test of Fisher. Towards this end, data were normalized to the mean values of the respective training group (i.e. hypoxia and normoxia) prior to training. Relationships between molecular, cellular and functional parameters were assessed with Pearson correlations. A cut-off of |*r*| ≥ 0.70 and *p* < 0.05 was applied. For factors where normal distribution could be rejected, a Spearman ranks correlation was applied.

## Results

### Participant characteristics

The anthropometric and exercise characteristics of the participants are given in Table 1. No differences were noted for any of these values between the participants of the normoxia and hypoxia group before training. The percentage of type I fibres did not differ between the normoxia (46.4 ± 2.6 %) and hypoxia (44.2 ± 4.4 %) group (*p* = 0.33).

**Table 1:** Subject characteristics: summary of the anthropometric and performance measures of the subjects of the normoxia and hypoxia-training group

		Age (years)	Height (cm)	BMI (m^2^/kg)	Fat (%)	Weight (kg)	Normoxia		Hypoxia	
							*V*O_2max_ (ml O_2_/min/kg)	*P*_max_ (W)	*V*O_2max_ (ml O_2_/min/kg)	*P*_max_ (W)
Normoxia training group										
Pre	Mean	29.0	179.7	24.6	15.0	78.9	43.4	298.2	33.7	240.8
	SE	2.6	2.4	2.1	4.4	5.6	9.5	52.8	9.2	44.3
Post	Mean			24.5	15.2	78.7	47.5	333.7	37.0	254.8
	SE			4.8	10.4	13.1	10.1	58.4	9.9	40.4
	∆			0 %	1 %	0 %	9 %	12 %	10 %	6 %
	*t* test			0.636	0.608	0.752	0.008	0.018	0.001	0.007
Hypoxia-training group										
Pre	Mean	25.8	169.4	23.5	14.4	67.8	43.6	282.4	36.7	220.0
	SE	2.2	2.2	1.3	2.9	5.4	3.1	21.3	1.5	14.4
Post	Mean			23.2	15.5	67.0	51.1	300.4	39.7	241.2
	SE			1.4	3.9	5.4	3.8	22.2	2.5	15.0
	∆			−1 %	8 %	−1 %	17 %	6 %	8 %	10 %
	*t* test			0.160	0.592	0.163	0.006	0.005	0.091	0.001
Interaction effect				0.467	0.650	0.493	0.226	0.014	0.879	0.096

*P* values of two-tailed *t* test are presented

### Molecular effects of single bout of exercise in hypoxia

Subjects performed an endurance exercise test under normoxia or hypoxia at an intensity matched for the same respective maximal aerobic power to assess the acute adaptive response to training. Exercise significantly lowered arterial oxygen saturation and increased serum lactate in both, the normoxia and hypoxia, training group (Table 2). Both the drop in arterial oxygen saturation and venous lactate concentration was accentuated in the hypoxia-training group emphasizing the high metabolic strain imposed during single bout of exercise in hypoxia. Both forms of training improved *V*O_2max_ and *P*_max_, but not whole body composition, and this was pronounced for the test condition corresponding to training stimulus (Table 1).

**Table 2:** Characteristics of the exercise stimulus

	Arterial oxygen saturation (%)	Lactate (mM)
Normoxia		
Pre-exercise	98.2 ± 0.5	1.7 ± 0.2
∆post-exercise	−2.2 ± 1.0	4.0 ± 0.9
*p* value	0.025	0.008
Hypoxia		
Pre-exercise	92.5 ± 1.1	1.8 ± 0.3
∆post-exercise	−15.2 ± 1.7	6.4 ± 0.7
*p* value	<0.001	<0.001
∆hypoxia vs. normoxia	<0.001	0.07

Subjects exercised on a bicycle ergometer for 30 min at 65 % *P*_max_ after 10 min of a warm-up at 40 %* P*_max_ in normoxia or hypoxia with an inspired oxygen fraction of 21 or 12 % at 560 m above sea level (the latter being equivalent to 4,000 m above sea level). Arterial oxygen saturation and venous lactate under the respective oxygenation was assessed at the fingertip at rest and after the endurance exercise test. *p* values of two-tailed *t* test are presented

**Fig. 2:**
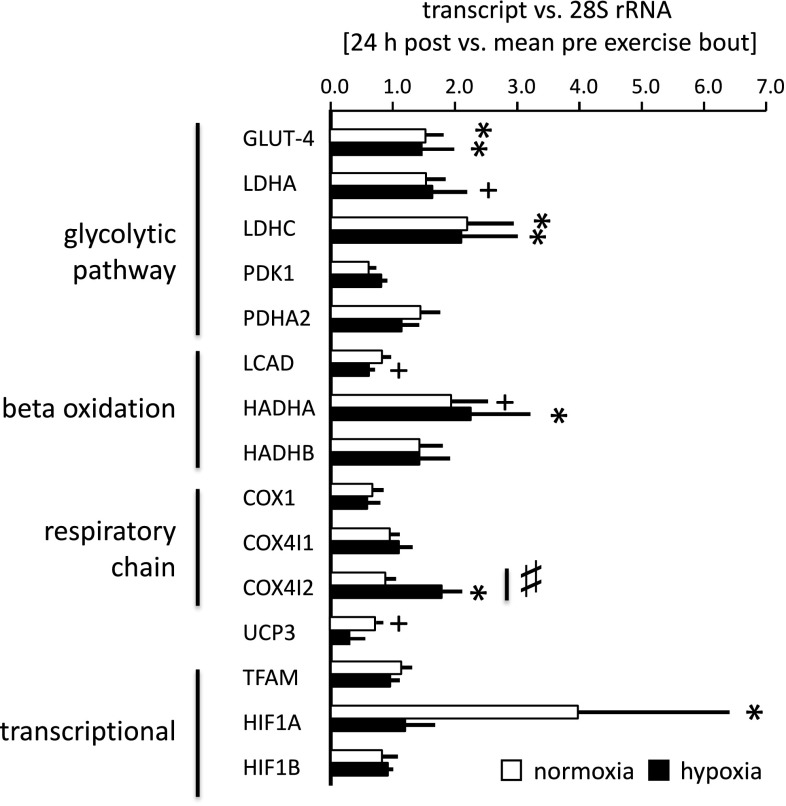
Hypoxia-specific transcript expression after the exercise bout. Bar graph visualizing the mean fold changes + standard error (SE) of 13 selected gene transcripts in *vastus lateralis* muscle of the untrained men 24 h into recovery from the bout of endurance exercise in normoxia or hypoxia, respectively, vs. pre-exercise levels. ^#^ *p* < 0.05 for the interaction effect between the repeated factor pre/post-exercise × co-stimulus (normoxia/hypoxia, repeated ANOVA, *n* = 5–6). *p < 0.05 and ^+^0.05 ≤ *p* < 0.10 for post vs. pre exercise differences (post hoc test of Fisher)

Figure 2 shows the changes in transcripts in *vastus lateralis* 24 h after a single bout of exercise in normoxia or hypoxia. Transcript expression of isoform 2 of cytochrome *c* oxidase subunit 4 (i.e. COX4I2), but none of the 14 other selected gene transcripts, demonstrated an interaction between ‘exercise (pre/24 h post)’ and ‘co-stimulus (normoxia/hypoxia)’; this interaction was visible as an increase in COX4I2 transcript levels 24 h after exercise in hypoxia but not in normoxia (Fig. 2).

### Hypoxia-related muscle adjustments with training

Subjects repeated the bicycle-type exercise under the respective co-stimulus, i.e. normoxia or hypoxia, 30 times during the following 6 weeks. Hypoxia enhanced the endurance training-induced increase in subsarcolemmal mitochondrial density in *vastus lateralis* muscle (Table 3). An interaction effect (*p* = 0.045) between training (pre/post) × co-stimulus (normoxia/hypoxia) on CSA of all muscle fibres was identified. The effect was explained by a reduction in CSA of type I (*p* = 0.037) and a trend for a reduction in the CSA of type II muscle fibres (*p* = 0.057) in the normoxia group with training (Table 3).

**Table 3:** Hypoxia-specific training-effects in skeletal muscle

	Normoxia			Hypoxia			
	Pre	Post	*p* value	Pre	Post	*p* value	Interaction effect
CSA all fibres (μm^2^)	6,517 ± 481	6,427 ± 383	0.010	7,554 ± 304	6,293 ± 529	0.828	0.045
CSA type I fibre (μm^2^)	6,648 ± 537	6,543 ± 523	0.037	7,909 ± 302	6,708 ± 544	0.851	0.166
CSA type II fibre (μm^2^)	6,385 ± 524	6,311 ± 308	0.057	7,198 ± 346.6	5,877 ± 559	0.913	0.198
Subsarcolemmal mitochondria (%)	0.72 ± 0.13	1.38 ± 0.19	0.004	0.79 ± 0.27	1.98 ± 0.16	0.001	0.017
Interfibrillar mitochondria (%)	3.57 ± 0.35	4.54 ± 0.33	0.023	3.88 ± 0.53	4.60 ± 0.33	0.031	0.937
Capillaries per fibre	1.83 ± 0.10	1.82 ± 0.11	0.601	1.75 ± 0.21	2.02 ± 0.26	0.086	0.318
Capillary density	528.3 ± 28.1	686.4 ± 60.3	0.238	602.1 ± 56.3	688.7 ± 45.4	0.010	0.240
DT (min)	4.25 ± 0.22	4.00 ± 0.29	0.11	3.40 ± 0.42	3.54 ± 0.33	0.69	0.34
ORT (s)	3.32 ± 0.26	3.87 ± 0.57	0.74	3.53 ± 0.54	2.58 ± 0.26	0.07	0.06
[Mb] (mmol/kg)	0.29 ± 0.01	0.29 ± 0.01	0.93	0.29 ± 0.01	0.28 ± 0.00	0.33	0.64
BOCR (ml O_2_/kg)	1.70 ± 0.14	1.80 ± 0.16	0.14	2.21 ± 0.28	2.00 ± 0.21	0.22	0.08

Median ± SE of selected endurance training-induced adjustments in vastus lateralis muscle and interaction effect with the co-stimulus (i.e. normoxia or hypoxia; repeated ANOVA with post hoc test of Fisher). The values reproduce previously published material in tabular form (Schmutz et al. 2010)

**Fig. 3:**
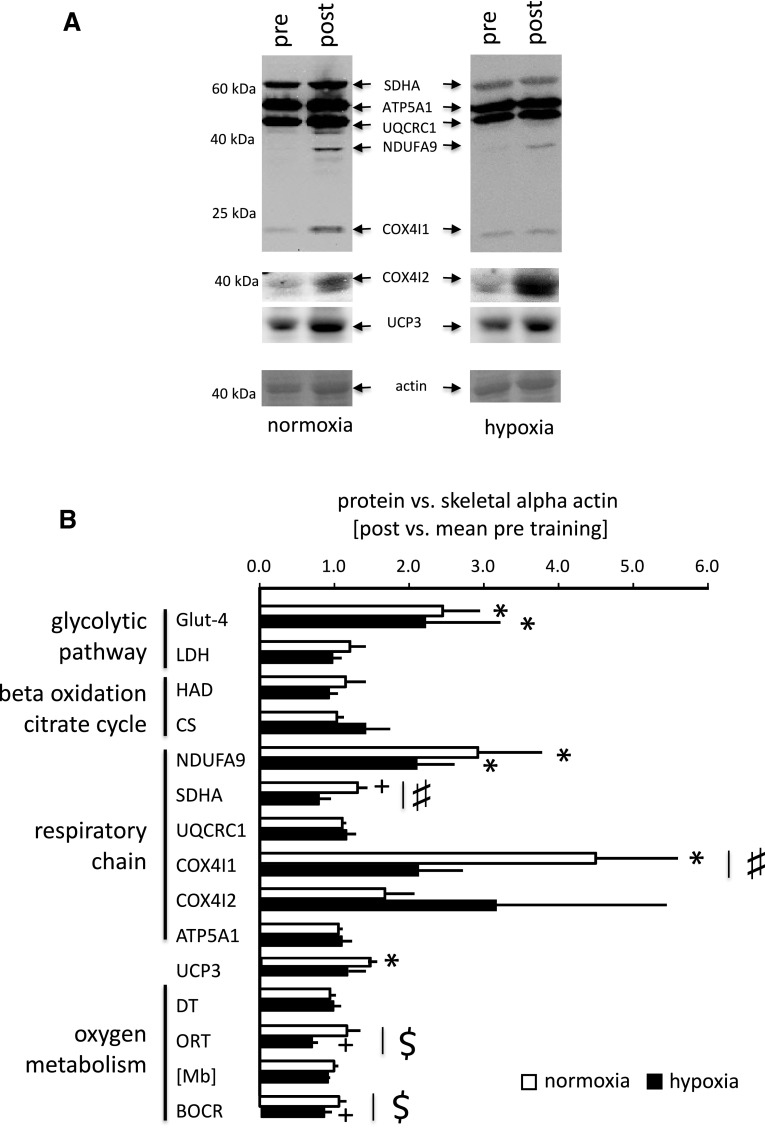
Hypoxia-modulated adaptations in muscle protein and oxygen metabolism to endurance training. **a** Examples of immunoblots for mitochondrial proteins in *vastus lateralis* muscle of a subject pre- and post-endurance training in normoxia and hypoxia, respectively. The abbreviated name and molecular weight of the detected proteins are indicated. The Ponceau S stained membrane before immunoblotting is shown below each blot to visualize protein loading based on the stained band corresponding to actin. **b** Bar graph visualizing the mean fold changes + SE of protein (activity) levels in *m. vastus lateralis* after 6 weeks of endurance training in normoxia (*n* = 6) or hypoxia (*n* = 5) training. ^#^ *p* < 0.05 and ^$^0.05 ≤ *p* < 0.10, respectively, for the interaction between the ‘pre/post-exercise’ ×  ‘co-stimulus’ (repeated ANOVA). * and ^+^ denote *p* < 0.05 for values post vs. pre training (post hoc test of Fisher)

Biochemical analysis of biopsy samples revealed a number of changes in protein expression levels and enzyme activities in the *vastus lateralis* muscle after endurance training in both normoxia and hypoxia (Fig. 3). Interactions between training and oxygen levels were evident for SDHA (*p* = 0.043) and COX4I1 (*p* = 0.045) protein. No such interaction was found for any of the other proteins or activities of mitochondrial enzymes (Fig. 3). Fold changes in COX4I1 protein were higher after training in normoxia than hypoxia and fold changes in SDHA protein tended to be selectively elevated after training in normoxia.

Trends for interactions were noted for ‘oxygen repletion time’ (ORT; *p* = 0.07) and ‘basal oxygen consumption rate’ (BOCR; *p* = 0.07) in *vastus* muscles (Fig. 3b). ORT and BOCR tended to be reduced after training in hypoxia.

### Association of muscle adjustments to single and repeated endurance exercise with mitochondrial plasticity

**Fig. 4:**
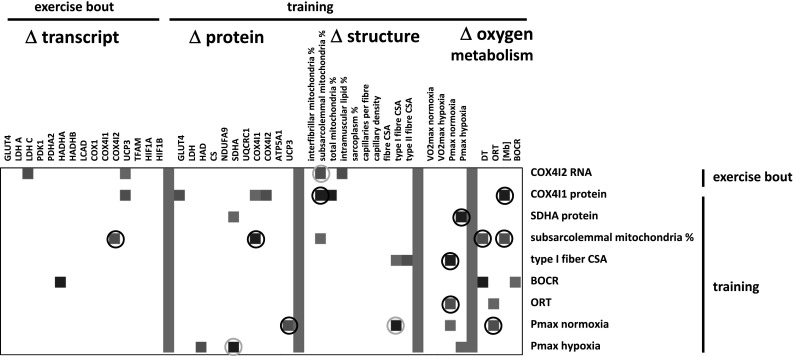
Interrelationships for hypoxia-specific adjustments with exercise. Matrix visualising the correlations between factors demonstrating hypoxia-specific regulation with single endurance exercise and training. Fold changes which significance exceeded the threshold of |*r*| > 0.7 and *p* < 0.05 are shown in colour coding (*red up*, *blue down*). *White colour* refers to values below the threshold for significance (|*r*| < 0.70). *Grey* symbolises suppressed values. Correlations that are deemed of interest are circled. Double represented relationships are once *circled in grey *(colour figure online)

A number of correlations were observed for muscle-related variables of aerobic metabolism, which demonstrated hypoxia-specific changes with endurance exercise/training (Fig. 4).

The major observations were that the training-induced changes in subsarcolemmal mitochondria volume density in *vastus lateralis* muscle with endurance training were positively correlated with level alterations in the COX4I2 transcript 24 h after the single bout of endurance exercise and the de-oxygenation time and myoglobin content after training (*r* ≥ 0.75). Conversely, level alterations in COX4I1 protein after training were negatively correlated with training-induced changes in subsarcolemmal mitochondria volume density. Level alterations in the COX4I2 transcript 24 h after single endurance exercise were positively correlated with those of LDHC, PDHA2, HADHA and peak aerobic performance in hypoxia after endurance training. Conversely, the latter changes in peak aerobic performance in hypoxia after training were negatively correlated with those in SDHA protein.

The training-induced alterations of SDHA (*r* = 0.70), and maximal aerobic performance in normoxia (*r* = 0.72) were correlated with the delta in arterial oxygen saturation after the first endurance exercise in the respective training condition. Training-induced changes in CS (*r* = 0.73) and COX4I2 (*r* = 0.83) protein were correlated to the delta in serum lactate after the first endurance exercise in the respective training condition.

## Discussion

Mitochondrial metabolism in skeletal muscle undergoes distinct quantitative improvements with repeated muscle work (endurance training). For instance, mitochondrial volume density and markers of mitochondrial metabolism in knee extensor muscle are typically elevated in previously untrained subjects by 20 % after 30 intense bicycle exercise sessions over 4–6 weeks (Bakkman et al. 2007; Hoppeler et al. 1985). The appearance of deoxymyoglobin is indicative for a drop in muscle oxygen tension with the onset of intense muscle work from 24 to 8 Torr, which is further accentuated when the fraction of inspired oxygen is 12 % (Flueck 2009; Richardson et al. 1995, 2001). It has been argued that the repeated reduction in oxygen tension in exercised muscle during training sessions with periods of recovery to normal oxygen tensions is an important part of the stimulus that drives muscle adaptations to endurance training (reviewed in Flueck 2009; Wagner 2012). The increased volume density of subsarcolemmal mitochondria, with an intrinsically lower rate of oxygen consumption than central (also being called intramyofibrillar) mitochondria (Adhihetty et al. 2003), in response to endurance training in hypoxia has been suggested to reflect an improved efficiency of cellular respiratory processes (reviewed by Desplanches et al. 1993; Schmutz et al. 2010; Vogt et al. 2001).

To test the molecular regulation of muscle respiration, we deployed an established endurance-training regime under the added co-stimulus of hypoxia. Adjustments of mitochondria-associated transcript and protein expression were probed and assessed for linear relationships to cellular and functional variables of oxygen metabolism. The choice of the 24-h time point to assess transcript expression after bicycle exercise was based on the established switch in COX4 isoform expression after 24 h of hypoxia exposure in culture (Fukuda et al. 2007), the responsiveness of transcript expression at this time point (Busso and Fluck 2013), and the fact that at this time point the next training session would normally take place. Our study identified hypoxia-specific regulation of the COX4I2 transcript after a single bout of exercise and found that the exercise-induced changes in this transcript also correlated to exercise-induced changes in the transcript for the mitochondria-associated isoform C of lactate dehydrogenase, LDHC, and training-induced changes in volume density of subsarcolemmal mitochondria (Fig. 4). Interestingly, the fold change of the encoded protein, COX4I2, after endurance training was correlated to the delta in serum lactate after the first endurance exercise in the respective training condition (*r* = 0.83). Conversely, no other assessed gene transcript demonstrated hypoxia-specific regulation post exercise. Our findings suggest that COX4I2 transcript up-regulation is a marker of hypoxia-induced mitochondrial plasticity.

To the best of our understanding, this is the first report commenting on the relation between hypoxia training-induced alterations in gene expression at the protein level with muscle composition and oxygen metabolism in men. We identify that hypoxia-specific effects on metabolic muscle plasticity are also reflected by interaction effects between the co-stimulus and training on levels of SDHA and COX4I1 protein and myoglobin-based variables of oxygen metabolism, ORT and BOCR (Fig. 3b). ORT is thought to reflect perfusion, whereby a shortened ORT is indicative of a faster perfusion (Slotboom et al. 1998). The trend for a hypoxia-specific reduction in ORT with 6 weeks of endurance training (Fig. 3b) is thus suggestive for improvements in muscle perfusion after hypoxia training. This is consistent with the increased capillary density and trend (*p* = 0.086) for an increase in the number of capillaries per fibre of vastus lateralis in the subjects with endurance training in hypoxia while both measures of capillarity were not altered with endurance training in normoxia (Table 3). The interaction regarding basal oxygen consumption rate (BOCR) is further support for hypoxia-specific changes in oxygen muscle metabolism induced by endurance training. To our understanding, these data are the first to document a drop in BOCR with training in hypoxia. We interpret these observations as support for the hypothesis that the efficiency of respiratory processes in skeletal muscle is particularly improved with a ‘living-low training-high’ paradigm.

Our findings identify that the employed endurance-training protocol in normoxia produced a reduction in the CSA of type I muscle fibres and increased UCP3 levels, which did not occur in hypoxia (Fig. 3b; Table 3). UCP3 content has been reported to demonstrate a negative association with work efficiency work (Mogensen et al. 2006). In contrast with the former findings, we identify positive and negative correlations, respectively, between the changes of UCP3 and CSA of type I muscle fibres with *P*_max_ with endurance training (Fig. 4). These observations suggest that adjustments in UCP3 levels, ORT, and type I fibre size are associated with the processes that set the scope of improvement in peak aerobic performance (*P*_max_) with endurance training.

Interestingly, we identify that the endurance training-induced fold change in volume density of subsarcolemmal mitochondrial was positively correlated to changes in myoglobin content (i.e. *r* = 0.74). This contrasts the observation that the distribution of subsarcolemmal mitochondria with respect to capillaries is largely independent from the level of myoglobin (Kayar et al. 1988). It has been reported that myoglobin concentration (termed content herein) is increased after one-legged endurance training in hypobaric conditions (Terrados et al. 1990). In our study, however, myoglobin content was not affected by endurance training in vastus muscles (Fig. 3b). Our study was not designed to explore the difference between the one-legged exercise of the latter study by Terrados et al. (1990) and our two-legged protocol. However, we assume that a less pronounced reduction in muscle oxygenation with one-legged exercise at simulated 2,300 m above sea level compared to our two-legged protocol during which exercise was performed at simulated 4,000 m above sea level would be a possible explanation for the observed difference. In this regard, the reciprocal, linear relationships between fold changes of COX4I1 (Fig. 4) and the other mitochondrial markers (ATP5A1 and UQCRC1, not shown) and myoglobin content (*r* < −0.70) is of special interest: it relates to the reported hypoxia sensitivity of adaptations to exercise at altitude where the best aerobically trained gain the least in maximal oxygen uptake (Angermann et al. 2006; Mollard et al. 2007).

A puzzling observation of our study was that the increase in SDHA protein with endurance training in normoxia (Fig. 4a) was blunted with endurance training in hypoxia (Fig. 3b) and correlated negatively with the fold changes in *P*_max_ (Fig. 4). SDH has been shown before to be associated with changes in subsarcolemmal mitochondria with endurance training (Chilibeck et al. 1998, 2002) and as an element of the Krebs cycle and respiratory complex II (Fig. 1b), SDHA, is a critical element in mitochondrial metabolism. Our findings are of interest with regard to the recognized contribution of respiratory complex II to reactive oxygen species production and the decrease in reactive oxygen species with the down-regulation of SDH activity of complex II in hypoxia-adapted flies (Ali et al. 2012; Quinlan et al. 2012). This is of interest given that SDHA, was the only protein which level alteration with endurance training correlated with the decline in arterial oxygen saturation during the Single bout of exercise (*r* = 0.70; Table 2). Our observations therefore raise the hypothesis that the down-regulation of myocellular SDHA after endurance training in hypoxia reflects a possible uncoupling of the Krebs cycle from adaptive changes in the respiration chain, which may minimize the production of reactive oxygen species.

Based on work in culture (Fukuda et al. 2007), it has been proposed that hypoxia promotes a switch in isoform expression for subunit 4 of the oxygen-sensitive complex IV of mitochondrial respiration towards isoform 2 (i.e. COX4I2) with elevated affinity for oxygen after exercise in hypoxia. This has been shown to manifest 24-h after exposure to hypoxia by increased expression of transcript and protein of COX4I2 when the related COX4I1 protein, being encoded on a different gene, is degraded (Fukuda et al. 2007). This view is comforted by our observation on the hypoxia-specific increase in transcript levels for COX4I2 24 h after the exercise bout (Fig. 2). This latter finding is compatible with our observations in intact human skeletal muscle, for which we identify a selective increase in COX4I1 protein after training in normoxia (Fig. 3b), which correlated negatively with changes in volume density of subsarcolemmal mitochondria (Fig. 4). By contrast, and as pointed out before the change in COX4I2 transcript post-exercise correlated positively with the changes in volume density of subsarcolemmal mitochondria with training (Fig. 4). However, we did not find an increase in COX4I2 protein in *vastus lateralis* muscle after endurance training in hypoxia (Fig. 3b). Retrospective power analysis identified that the effect of hypoxia on the endurance-training effect would have been resolved at a number of 12 biological replicas (or subjects) for COX4I2 protein. We interpret these observations to reflect a burst in mitochondrial biogenesis post-endurance exercise which connects the increase in COX4I2 mRNA after endurance exercise in hypoxia to the addition of new mitochondria under the sarcolemma with 30 repetitions of the exercise stimulus (Adhihetty et al. 2003). In conjunction we identified a linear correspondence between changes in the volume density of subsarcolemmal mitochondria and de-oxygenation time (DT) and myoglobin content ([Mb], *r* > 0.70). The observed associations support the proposition on a role of the subsarcolemmal population of mitochondria in the extraction of myocellular oxygen (Kayar et al. 1988).

A number of limitations apply to our study. Foremost this relates to the low number of biological replicas of our study, which was due to ethical considerations and labour-intensive work for an invasive study with human volunteers. Therefore, certain correlations may also be suspected to represent a statistical type I error. However, many of the identified correlations are predicted (for example, type I fibre CSA: type II fibre CSA) and the displayed correlations with |*r*| values >0.7 indicate a large effect size. As well it is possible that due to the selection of a 24-h time point after the single exercise bout, certain transcript level alterations that occur earlier may be missed (Pilegaard et al. 2003). Then we identify that we have no data on muscle oxygenation during the single bout of exercise. Muscle oxygenation has been assessed in idealized situations of single-leg exercise based on the dMb signal (Richardson et al. 1995, 2001) but this was no option because of the difficulty of performing strong two-legged exercise in a MR scanner and recording the low SNR signal of dMb dynamically at the same time. However, we do find correlations between parameters of oxygen transients in skeletal muscle as quantified at rest by ^1^H-MRS prior and post-endurance training (i.e. BOCR, DT, [Mb], ORT), with muscle capillarity, mean muscle fibre area and arterial oxygen saturation post exercise (data shown). These relationships establish that cardiovascular parameters of oxygen flux during dynamic exercise are matched to the functional capacity for oxygen uptake in peripheral skeletal muscle. The findings support the relevance of our approach to assess the relationship between muscle reactions (i.e. gene expression) and training-induced alterations in muscle respiration from changes in muscle oxygenation at rest.

## Conclusion

The findings support the notion that endurance exercise in hypoxia promotes the biogenesis of the subsarcolemmal pool of mitochondria via a switch in the expression of cytochrome *c* oxidase subunit 4 isoforms and an uncoupling between the Krebs cycle and the respiratory chain at the level of SDH. These observations and the trend of effect on basal oxygen consumption rate are of specific interest given the spurt of interest for hypoxia-training paradigms to combat metabolic diseases (Wiesner et al. 2010). ^1^H-MRS of deoxymyoglobin is suggested as tool to resolve local improvements in oxygen metabolism with endurance training in hypoxia.

## Abbreviations

ATP5A1: Mitochondrial ATP synthase subunit alpha
BOCR: Basal oxygen consumption rate
COX1: Cytochrome *c* oxidase subunit 1
COX4I1: Isoform 1 of cytochrome *c* oxidase subunit 4
COX4I2: Isoform 2 of cytochrome *c* oxidase subunit 4
CS: Citrate synthase
CSA: Cross-sectional area
DT: De-oxygenation time
GLUT4: Facilitative glucose transporter 4
HADH: 3-Hydroxyacyl-CoA dehydrogenase
HADHA: An isoform of 3-hydroxyacyl-CoA dehydrogenase
HIF1A: Subunit alpha of hypoxia-inducible factor 1
HIF1B: Subunit beta of hypoxia-inducible factor 1
LCAD: Long-chain-specific acyl-CoA dehydrogenase
LDH A: Isoform A of lactate dehydrogenase
LDHB: Isoform B of lactate dehydrogenase
LDHC: Isoform C of lactate dehydrogenase
[Mb]: Myoglobin content
NDUFA9: NADH dehydrogenase (ubiquinone) 1 alpha subcomplex subunit 9
ORT: Oxygen repletion time
PDHA2: Subunit A of pyruvate dehydrogenase E1 component
PDK1: Pyruvate dehydrogenase kinase isoenzyme 1
*P*_max_: Peak aerobic power output
SaO_2_: Arterial oxygen saturation
SDH: Succinate dehydrogenase
TFAM: Mitochondrial transcription factor A
UCP3: Uncoupling protein 3
UQCRC1: Subunit 1 of cytochrome *b*-*c*1 complex
*V*O_2max_: Maximal oxygen uptake

## References

[CR1] Adhihetty PJ, Irrcher I, Joseph AM, Ljubicic V, Hood DA (2003). Plasticity of skeletal muscle mitochondria in response to contractile activity. Exp Physiol.

[CR2] Ali SS, Hsiao M, Zhao HW, Dugan LL, Haddad GG, Zhou D (2012). Hypoxia-adaptation involves mitochondrial metabolic depression and decreased ROS leakage. PLoS ONE.

[CR3] Andrade FH, McMullen CA (2006). Lactate is a metabolic substrate that sustains extraocular muscle function. Pflugers Arch.

[CR4] Angermann M, Hoppeler H, Wittwer M, Däpp C, Howald H, Vogt M (2006). Effect of acute hypoxia on maximal oxygen uptake and maximal performance during leg and upper-body exercise in Nordic combined skiers. Int J Sports Med.

[CR5] Bakkman L, Sahlin K, Holmberg HC, Tonkonogi M (2007). Quantitative and qualitative adaptation of human skeletal muscle mitochondria to hypoxic compared with normoxic training at the same relative work rate. Acta Physiol (Oxf).

[CR6] Baumgartner I, Thoeny HC, Kummer O, Roefke C, Skjelsvik C, Boesch C, Kreis R (2005). Leg ischemia: assessment with MR angiography and spectroscopy. Radiology.

[CR7] Busso T, Fluck M (2013). A mixed-effects model of the dynamic response of muscle gene transcript expression to endurance exercise. Eur J Appl Physiol.

[CR8] Carlier PG, Bertoldi D, Baligand C, Wary C, Fromes Y (2006). Muscle blood flow and oxygenation measured by NMR imaging and spectroscopy. NMR Biomed.

[CR9] Chilibeck PD, Bell GJ, Socha T, Martin T (1998). The effect of aerobic exercise training on the distribution of succinate dehydrogenase activity throughout muscle fibres. Can J Appl Physiol.

[CR10] Chilibeck PD, Syrotuik DG, Bell GJ (2002). The effect of concurrent endurance and strength training on quantitative estimates of subsarcolemmal and intermyofibrillar mitochondria. Int J Sports Med.

[CR11] Desplanches D, Mayet MH, Sempore B, Flandrois R (1987). Structural and functional responses to prolonged hindlimb suspension in rat muscle. J Appl Physiol.

[CR12] Desplanches D, Hoppeler H, Linossier MT, Denis C, Claassen H, Dormois D, Lacour JR, Geyssant A (1993). Effects of training in normoxia and normobaric hypoxia on human muscle ultrastructure. Pflugers Arch.

[CR01] Durieux AC, D'Antona G, Desplanches D, Freyssenet D, Klossner S, Bottinelli R, Flück M (2009) Focal adhesion kinase is a load-dependent governor of the slow contractile and oxidative muscle phenotype. J Physiol 587(Pt 14): 3703–3717 10.1113/jphysiol.2009.171355 PMC2742292 19470782

[CR13] Elustondo PA, White AE, Hughes ME, Brebner K, Pavlov E, Kane DA (2013). Physical and functional association of lactate dehydrogenase (LDH) with skeletal muscle mitochondria. J Biol Chem.

[CR14] Fluck M (2006). Functional, structural and molecular plasticity of mammalian skeletal muscle in response to exercise stimuli. J Exp Biol.

[CR15] Fluckey JD, Ploug D, Galbo H (1999). Mechanisms associated with hypoxia- and contraction-mediated glucose transport in muscle are fibre-dependent. Acta Physiol Scand.

[CR16] Flueck M (2009). Plasticity of the muscle proteome to exercise at altitude. High Alt Med Biol.

[CR17] Flueck M, Eyeang-Bekale N, Heraud A, Girard A, Gimpl M, Seynnes OR, Rittweger J, Niebauer J, Mueller E, Narici M (2011). Load-sensitive adhesion factor expression in the elderly with skiing: relation to fiber type and muscle strength. Scand J Med Sci Sports.

[CR18] Fukuda R, Zhang H, Kim JW, Shimoda L, Dang CV, Semenza GL (2007). HIF-1 regulates cytochrome oxidase subunits to optimize efficiency of respiration in hypoxic cells. Cell.

[CR19] Giraud MN, Fluck M, Zuppinger C, Suter TM (2005). Expressional reprogramming of survival pathways in rat cardiocytes by neuregulin-1beta. J Appl Physiol.

[CR20] Green H, Burnett M, Smith I, Tupling S, Ranney D (2009). Failure of hypoxia to exaggerate the metabolic stress in working muscle following short-term training. Am J Physiol.

[CR21] Hoppeler H, Desplanches D (1992). Muscle structural modifications in hypoxia. Int J Sports Med.

[CR22] Hoppeler H, Howald H, Conley K, Lindstedt SL, Claassen H, Vock P, Weibel ER (1985). Endurance training in humans: aerobic capacity and structure of skeletal muscle. J Appl Physiol.

[CR23] Horowitz JF, Kaufman AE, Fox AK, Harber MP (2005). Energy deficit without reducing dietary carbohydrate alters resting carbohydrate oxidation and fatty acid availability. J Appl Physiol.

[CR24] Kayar SR, Hoppeler H, Essen-Gustavsson B, Schwerzmann K (1988). The similarity of mitochondrial distribution in equine skeletal muscles of differing oxidative capacity. J Exp Biol.

[CR25] Kreis R, Bruegger K, Skjelsvik C, Zwicky S, Ith M, Jung B, Baumgartner I, Boesch C (2001). Quantitative (1)H magnetic resonance spectroscopy of myoglobin de- and reoxygenation in skeletal muscle: reproducibility and effects of location and disease. Magn Reson Med.

[CR26] Mogensen M, Bagger M, Pedersen PK, Fernstrom M, Sahlin K (2006). Cycling efficiency in humans is related to low UCP3 content and to type I fibres but not to mitochondrial efficiency. J Physiol.

[CR27] Mollard P, Woorons X, Letournel M, Lamberto C, Favret F, Pichon A, Beaudry M, Richalet JP (2007). Determinants of maximal oxygen uptake in moderate acute hypoxia in endurance athletes. Eur J Appl Physiol.

[CR28] Perry CG, Lally J, Holloway GP, Heigenhauser GJ, Bonen A, Spriet LL (2010). Repeated transient mRNA bursts precede increases in transcriptional and mitochondrial proteins during training in human skeletal muscle. J Physiol.

[CR29] Pilegaard H, Ordway GA, Saltin B, Neufer PD (2000). Transcriptional regulation of gene expression in human skeletal muscle during recovery from exercise. Am J Physiol.

[CR30] Pilegaard H, Saltin B, Neufer PD (2003). Exercise induces transient transcriptional activation of the PGC-1alpha gene in human skeletal muscle. J Physiol.

[CR31] Ponsot E, Dufour SP, Zoll J, Doutrelau S, N’Guessan B, Geny B, Hoppeler H, Lampert E, Mettauer B, Ventura-Clapier R, Richard R (2006). Exercise training in normobaric hypoxia in endurance runners. II. Improvement of mitochondrial properties in skeletal muscle. J Appl Physiol.

[CR32] Quinlan CL, Orr AL, Perevoshchikova IV, Treberg JR, Ackrell BA, Brand MD (2012). Mitochondrial complex II can generate reactive oxygen species at high rates in both the forward and reverse reactions. J Biol Chem.

[CR33] Richardson RS, Noyszewski EA, Kendrick KF, Leigh JS, Wagner PD (1995). Myoglobin O_2_ desaturation during exercise. Evidence of limited O_2_ transport. J Clin Invest.

[CR34] Richardson RS, Newcomer SC, Noyszewski EA (2001). Skeletal muscle intracellular PO_2_ assessed by myoglobin desaturation: response to graded exercise. J Appl Physiol.

[CR35] Schmutz S, Dapp C, Wittwer M, Vogt M, Hoppeler H, Fluck M (2006). Endurance training modulates the muscular transcriptome response to acute exercise. Pflugers Arch.

[CR36] Schmutz S, Dapp C, Wittwer M, Durieux AC, Mueller M, Weinstein F, Vogt M, Hoppeler H, Flueck M (2010). A hypoxia complement differentiates the muscle response to endurance exercise. Exp Physiol.

[CR37] Scott GR, Egginton S, Richards JG, Milsom WK (2009). Evolution of muscle phenotype for extreme high altitude flight in the bar-headed goose. Proc Biol Sci.

[CR38] Slotboom J, Boesch C, Kreis R (1998). Versatile frequency domain fitting using time domain models and prior knowledge. Magn Reson Med.

[CR39] Terrados N, Jansson E, Sylvén C, Kaijser L (1990). Is hypoxia a stimulus for synthesis of oxidative enzymes and myoglobin?. J Appl Physiol.

[CR40] Tevald MA, Lanza IR, Befroy DE, Kent-Braun JA (2009). Intramyocellular oxygenation during ischemic muscle contractions in vivo. Eur J Appl Physiol.

[CR41] Vogt M, Puntschart A, Geiser J, Zuleger C, Billeter R, Hoppeler H (2001). Molecular adaptations in human skeletal muscle to endurance training under simulated hypoxic conditions. J Appl Physiol.

[CR42] Wagner PD (2012). Muscle intracellular oxygenation during exercise: optimization for oxygen transport, metabolism, and adaptive change. Eur J Appl Physiol.

[CR43] Wiesner S, Haufe S, Engeli S, Mutschler H, Haas U, Luft FC, Jordan J (2010). Influences of normobaric hypoxia training on physical fitness and metabolic risk markers in overweight to obese subjects. Obesity (Silver Spring).

[CR44] Zoll J, Ponsot E, Dufour S, Doutreleau S, Ventura-Clapier R, Vogt M, Hoppeler H, Richard R, Fluck M (2006). Exercise training in normobaric hypoxia in endurance runners. III. Muscular adjustments of selected gene transcripts. J Appl Physiol.

